# 4-[Bis(4-fluoro­phen­yl)meth­yl]piperazin-1-ium 2-hy­droxy­benzoate 2-hy­droxy­benzoic acid monosolvate

**DOI:** 10.1107/S1600536812012329

**Published:** 2012-03-24

**Authors:** A. S. Dayananda, H. S. Yathirajan, Ulrich Flörke

**Affiliations:** a Department of Studies in Chemistry, University of Mysore, Manasagangotri, Mysore 570 006, India; b Department Chemie, Fakultät für Naturwissenschaften, Universität Paderborn, Warburgerstrasse 100, D-33098 Paderborn, Germany

## Abstract

The title compound, C_17_H_19_F_2_N_2_^+^·C_7_H_5_O_3_^−^·C_7_H_6_O_3_, is a co-crystal from 4-[bis­(4-fluoro­phen­yl)meth­yl]piperazin-1-ium, salicylate anion and salicylic acid in a 1:1:1 ratio. In addition to an intra­molecular O—H⋯O hydrogen bond, the crystal packing shows hydrogen bonds between the piperazinium cation and salicylate anion (N—H⋯O), as well as between the salicylic acid mol­ecule and anion (O—H⋯O), giving rise to a three-dimensional network.

## Related literature

For the biological activity of piperazines, see: Bogatcheva *et al.* (2006 ▶); Brockunier *et al.* (2004 ▶). For related structures, see: Betz *et al.* (2011*a* ▶,*b* ▶); Fun *et al.* (2011 ▶); Jebamony & Thomas Muthiah (1998 ▶).
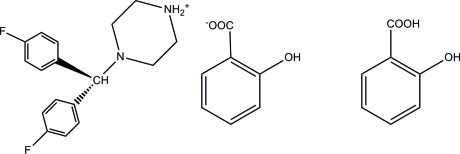


## Experimental

### 

#### Crystal data

C_17_H_19_F_2_N_2_^+^·C_7_H_5_O_3_^−^·C_7_H_6_O_3_
*M* *_r_* = 564.57
Monoclinic, 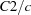

*a* = 33.157 (4) Å
*b* = 10.3007 (14) Å
*c* = 20.105 (3) Å
β = 124.447 (2)°
*V* = 5662.6 (13) Å^3^
*Z* = 8
Mo *K*α radiation
μ = 0.10 mm^−1^
*T* = 130 K
0.39 × 0.37 × 0.20 mm

#### Data collection

Bruker SMART APEX diffractometer
Absorption correction: multi-scan (*SADABS*; Sheldrick, 2004 ▶) *T*_min_ = 0.962, *T*_max_ = 0.980
26180 measured reflections
6761 independent reflections
4481 reflections with *I* > 2σ(*I*)
*R*_int_ = 0.045

#### Refinement

*R*[*F* ^2^ > 2σ(*F* ^2^)] = 0.047
*wR*(*F* ^2^) = 0.138
*S* = 0.86
6761 reflections
373 parameters
H-atom parameters constrained
Δρ_max_ = 0.27 e Å^−3^
Δρ_min_ = −0.21 e Å^−3^

### 

Data collection: *SMART* (Bruker, 2002 ▶); cell refinement: *SAINT* (Bruker, 2002 ▶); data reduction: *SAINT*; program(s) used to solve structure: *SHELXTL* (Sheldrick, 2008 ▶); program(s) used to refine structure: *SHELXTL*; molecular graphics: *SHELXTL*; software used to prepare material for publication: *SHELXTL* and local programs.

## Supplementary Material

Crystal structure: contains datablock(s) I, global. DOI: 10.1107/S1600536812012329/bt5853sup1.cif

Structure factors: contains datablock(s) I. DOI: 10.1107/S1600536812012329/bt5853Isup2.hkl

Supplementary material file. DOI: 10.1107/S1600536812012329/bt5853Isup3.cml

Additional supplementary materials:  crystallographic information; 3D view; checkCIF report

## Figures and Tables

**Table 1:** Hydrogen-bond geometry (Å, °)

*D*—H⋯*A*	*D*—H	H⋯*A*	*D*⋯*A*	*D*—H⋯*A*
N2—H2*C*⋯O22^i^	0.92	2.00	2.892 (2)	162
N2—H2*D*⋯O21	0.92	1.87	2.742 (2)	158
O12—H12⋯O22	0.84	1.73	2.5654 (18)	179
O13—H13⋯O11	0.84	1.83	2.574 (2)	146
O23—H23⋯O21	0.84	1.80	2.544 (2)	146

Symmetry code: (i) 
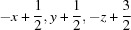
.

**Fig. 1.**
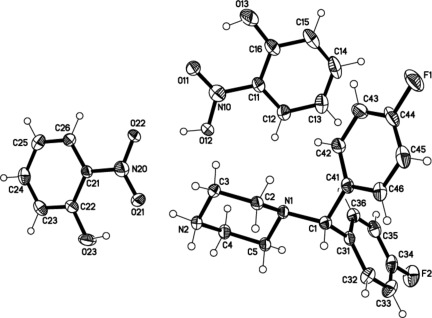
Molecular structure with displacement ellipsoids drawn at the 50% probability level.

## supplementary crystallographic information

### Comment

Piperazines are among the most important building blocks in today's drug discovery and are found in biologically active compounds across a number of different therapeutic areas (Brockunier *et al.*, 2004; Bogatcheva *et al.*, 2006). The crystal structures of 8-hydroxyquinolinium-salicylate-salicylic acid (Jebamony & Thomas Muthiah, 1998), 4-[bis(4-fluorophenyl)methyl]piperazin-1-ium 2-(2-phenylethyl) benzoate (Betz *et al.*, 2011*a*), 4-[bis(4-fluorophenyl)methyl]piperazin-1-ium picrate (Betz *et al.*, 2011*b*) and cyclobenzaprinium salicylate (Fun *et al.*, 2011) have been reported. In the course of our studies on the salts of piperazines and in view of the importance of piperazines, we now report the crystal and molecular structure of the title compound. The molecular structure of the cation is well known from ABADAK (Betz *et al.*, 2011*a*) and AZOTOZ (Betz *et al.*, 2011*b*). The packing is stabilized from intermolecular N2—H2C···O22(-*x* + 0.5, *y* + 0.5, -*z* + 1.5), N2—H2D···O21, O12—H···O22 interactions, intra-molecular H bonds are O13—H···O11 (salicylic acid) and O23—H···O21 (anion).

### Experimental

4,4'-Difluorobenzhydryl piperazine was obtained from *R. L*. Fine Chem., Bengaluru, India. 4,4'-Difluorobenzhydryl piperazine (2.88 g, 0.01 mol) was dissolved in 10 ml of ethanol and salicylic acid (1.38 g, 0.01 mol) was also dissolved in 10 ml of ethanol. Both the solutions were mixed and stirred in a beaker at 333 K for 30 minutes. The mixture was kept aside for a day at room temperature. The salt formed was filtered & dried in vaccum desiccator over phosphorous pentoxide. The compound was recrystallized from toluene by slow evaporation (m.p.: 408–413 K).

### Refinement

H atoms were clearly identified in difference syntheses, idealized and refined riding on the C/N parent atoms with C—H = 0.95 (aromatic) - 1.00 Å, N—H = 0.92 Å and with isotropic displacement parameters *U*_iso_(H) = 1.2U(C/N_eq_). Hydroxyl H atoms were refined with HFIX 147, O—H 0.84 Å and *U*_iso_(H) = 1.5U(O_eq_)

### Crystal data

**Table:**

C_17_H_19_F_2_N_2_^+^·C_7_H_5_O_3_^−^·C_7_H_6_O_3_	*F*(000) = 2368
*M**_r_* = 564.57	*D*_x_ = 1.324 Mg m^−^^3^
Monoclinic, *C*2/*c*	Mo *K*α radiation, λ = 0.71073 Å
Hall symbol: -C 2yc	Cell parameters from 3289 reflections
*a* = 33.157 (4) Å	θ = 2.2–21.4°
*b* = 10.3007 (14) Å	µ = 0.10 mm^−^^1^
*c* = 20.105 (3) Å	*T* = 130 K
β = 124.447 (2)°	Prism, colourless
*V* = 5662.6 (13) Å^3^	0.39 × 0.37 × 0.20 mm
*Z* = 8	

### Data collection

**Table:**

Bruker SMART APEX diffractometer	6761 independent reflections
Radiation source: sealed tube	4481 reflections with *I* > 2σ(*I*)
Graphite monochromator	*R*_int_ = 0.045
φ and ω scans	θ_max_ = 27.9°, θ_min_ = 1.5°
Absorption correction: multi-scan (*SADABS*; Sheldrick, 2004)	*h* = −43→43
*T*_min_ = 0.962, *T*_max_ = 0.980	*k* = −13→13
26180 measured reflections	*l* = −26→23

### Refinement

**Table:**

Refinement on *F*^2^	Primary atom site location: structure-invariant direct methods
Least-squares matrix: full	Secondary atom site location: difference Fourier map
*R*[*F*^2^ > 2σ(*F*^2^)] = 0.047	Hydrogen site location: difference Fourier map
*wR*(*F*^2^) = 0.138	H-atom parameters constrained
*S* = 0.86	*w* = 1/[σ^2^(*F*_o_^2^) + (0.0737*P*)^2^ + 5.3951*P*] where *P* = (*F*_o_^2^ + 2*F*_c_^2^)/3
6761 reflections	(Δ/σ)_max_ < 0.001
373 parameters	Δρ_max_ = 0.27 e Å^−^^3^
0 restraints	Δρ_min_ = −0.21 e Å^−^^3^

### Special details

**Table:**

Geometry. All e.s.d.'s (except the e.s.d. in the dihedral angle between two l.s. planes) are estimated using the full covariance matrix. The cell e.s.d.'s are taken into account individually in the estimation of e.s.d.'s in distances, angles and torsion angles; correlations between e.s.d.'s in cell parameters are only used when they are defined by crystal symmetry. An approximate (isotropic) treatment of cell e.s.d.'s is used for estimating e.s.d.'s involving l.s. planes.
Refinement. Refinement of *F*^2^ against ALL reflections. The weighted *R*-factor *wR* and goodness of fit *S* are based on *F*^2^, conventional *R*-factors *R* are based on *F*, with *F* set to zero for negative *F*^2^. The threshold expression of *F*^2^ > σ(*F*^2^) is used only for calculating *R*-factors(gt) *etc*. and is not relevant to the choice of reflections for refinement. *R*-factors based on *F*^2^ are statistically about twice as large as those based on *F*, and *R*- factors based on ALL data will be even larger.

### Fractional atomic coordinates and isotropic or equivalent isotropic displacement parameters (Å^2^)

**Table:**

	*x*	*y*	*z*	*U*_iso_*/*U*_eq_	
F1	−0.02784 (6)	0.43978 (13)	0.29760 (8)	0.0704 (5)	
F2	0.09200 (5)	1.15292 (13)	0.19161 (7)	0.0558 (4)	
N1	0.13141 (5)	0.87077 (13)	0.50424 (8)	0.0238 (3)	
N2	0.20766 (5)	0.90714 (14)	0.67270 (8)	0.0280 (3)	
H2C	0.1956	0.9716	0.6886	0.034*	
H2D	0.2381	0.8841	0.7166	0.034*	
C1	0.08253 (6)	0.89679 (16)	0.42988 (10)	0.0252 (4)	
H1A	0.0643	0.9543	0.4444	0.030*	
C2	0.12578 (6)	0.82397 (16)	0.56757 (10)	0.0263 (4)	
H2A	0.1049	0.7454	0.5482	0.032*	
H2B	0.1091	0.8913	0.5789	0.032*	
C3	0.17451 (6)	0.79218 (16)	0.64442 (10)	0.0281 (4)	
H3A	0.1695	0.7659	0.6866	0.034*	
H3B	0.1898	0.7185	0.6347	0.034*	
C4	0.21198 (6)	0.95705 (17)	0.60753 (10)	0.0290 (4)	
H4A	0.2281	0.8911	0.5943	0.035*	
H4B	0.2325	1.0363	0.6264	0.035*	
C5	0.16196 (6)	0.98805 (16)	0.53291 (10)	0.0263 (4)	
H5A	0.1462	1.0561	0.5456	0.032*	
H5B	0.1653	1.0215	0.4901	0.032*	
C31	0.08625 (6)	0.96641 (16)	0.36695 (10)	0.0259 (4)	
C32	0.06248 (7)	1.08357 (19)	0.33563 (11)	0.0345 (4)	
H32A	0.0449	1.1213	0.3551	0.041*	
C33	0.06396 (7)	1.1470 (2)	0.27582 (12)	0.0415 (5)	
H33A	0.0473	1.2269	0.2538	0.050*	
C34	0.08997 (7)	1.0914 (2)	0.24974 (11)	0.0370 (5)	
C35	0.11481 (7)	0.9764 (2)	0.28002 (11)	0.0362 (4)	
H35A	0.1332	0.9411	0.2614	0.043*	
C36	0.11238 (7)	0.91335 (18)	0.33841 (11)	0.0319 (4)	
H36A	0.1288	0.8328	0.3593	0.038*	
C41	0.05368 (6)	0.77155 (17)	0.39516 (10)	0.0275 (4)	
C42	0.07650 (7)	0.65443 (17)	0.40271 (11)	0.0338 (4)	
H42A	0.1111	0.6511	0.4311	0.041*	
C43	0.04914 (8)	0.54192 (19)	0.36909 (12)	0.0431 (5)	
H43A	0.0645	0.4613	0.3738	0.052*	
C44	−0.00086 (9)	0.5509 (2)	0.32877 (12)	0.0473 (6)	
C45	−0.02476 (8)	0.6645 (2)	0.31923 (12)	0.0449 (5)	
H45A	−0.0594	0.6672	0.2904	0.054*	
C46	0.00301 (7)	0.7753 (2)	0.35278 (11)	0.0350 (4)	
H46A	−0.0128	0.8556	0.3468	0.042*	
O11	0.26379 (5)	0.42053 (13)	0.65952 (8)	0.0436 (4)	
O12	0.26128 (5)	0.62897 (12)	0.62845 (8)	0.0369 (3)	
H12	0.2797	0.6354	0.6789	0.055*	
O13	0.21326 (9)	0.25463 (17)	0.54577 (11)	0.0899 (7)	
H13	0.2307	0.2818	0.5935	0.135*	
C10	0.24981 (7)	0.50656 (17)	0.60834 (11)	0.0309 (4)	
C11	0.21896 (6)	0.48031 (18)	0.52110 (11)	0.0319 (4)	
C12	0.20593 (7)	0.5776 (2)	0.46407 (11)	0.0357 (4)	
H12A	0.2171	0.6638	0.4816	0.043*	
C13	0.17710 (7)	0.5505 (2)	0.38262 (12)	0.0450 (5)	
H13A	0.1685	0.6175	0.3443	0.054*	
C14	0.16096 (9)	0.4253 (3)	0.35743 (14)	0.0609 (7)	
H14A	0.1410	0.4064	0.3014	0.073*	
C15	0.17332 (11)	0.3276 (3)	0.41204 (16)	0.0743 (9)	
H15A	0.1621	0.2416	0.3937	0.089*	
C16	0.20226 (9)	0.3541 (2)	0.49448 (14)	0.0554 (6)	
O21	0.30573 (5)	0.85892 (12)	0.77852 (8)	0.0380 (3)	
O22	0.31737 (5)	0.64556 (11)	0.78271 (7)	0.0335 (3)	
O23	0.37289 (5)	1.00850 (13)	0.88348 (10)	0.0485 (4)	
H23	0.3447	0.9876	0.8446	0.073*	
C20	0.33227 (6)	0.75940 (16)	0.80967 (10)	0.0272 (4)	
C21	0.38365 (6)	0.77746 (16)	0.88016 (10)	0.0256 (4)	
C22	0.40114 (7)	0.90104 (17)	0.91386 (11)	0.0314 (4)	
C23	0.44917 (7)	0.9159 (2)	0.98053 (12)	0.0381 (5)	
H23B	0.4610	0.9997	1.0030	0.046*	
C24	0.47949 (7)	0.8101 (2)	1.01393 (12)	0.0400 (5)	
H24A	0.5120	0.8208	1.0601	0.048*	
C25	0.46300 (7)	0.6875 (2)	0.98067 (12)	0.0380 (5)	
H25A	0.4841	0.6146	1.0035	0.046*	
C26	0.41582 (7)	0.67260 (17)	0.91448 (11)	0.0306 (4)	
H26A	0.4048	0.5887	0.8915	0.037*	

### Atomic displacement parameters (Å^2^)

**Table:**

	*U*^11^	*U*^22^	*U*^33^	*U*^12^	*U*^13^	*U*^23^
F1	0.0875 (10)	0.0534 (8)	0.0425 (7)	−0.0436 (8)	0.0201 (7)	−0.0115 (6)
F2	0.0613 (8)	0.0717 (9)	0.0370 (7)	−0.0058 (7)	0.0295 (6)	0.0173 (6)
N1	0.0243 (7)	0.0247 (7)	0.0190 (7)	−0.0016 (6)	0.0102 (6)	0.0004 (5)
N2	0.0287 (8)	0.0272 (7)	0.0188 (7)	0.0012 (6)	0.0079 (6)	0.0005 (6)
C1	0.0269 (9)	0.0257 (8)	0.0221 (8)	0.0015 (7)	0.0134 (7)	0.0001 (6)
C2	0.0296 (9)	0.0250 (8)	0.0219 (8)	−0.0019 (7)	0.0132 (7)	−0.0005 (7)
C3	0.0335 (9)	0.0244 (8)	0.0224 (8)	−0.0003 (7)	0.0133 (8)	0.0020 (7)
C4	0.0276 (9)	0.0308 (9)	0.0232 (8)	−0.0037 (7)	0.0112 (7)	0.0004 (7)
C5	0.0276 (9)	0.0263 (8)	0.0208 (8)	−0.0039 (7)	0.0111 (7)	0.0004 (7)
C31	0.0226 (8)	0.0293 (9)	0.0191 (8)	−0.0023 (7)	0.0078 (7)	−0.0013 (7)
C32	0.0316 (10)	0.0382 (10)	0.0322 (10)	0.0060 (8)	0.0171 (8)	0.0085 (8)
C33	0.0377 (11)	0.0425 (11)	0.0383 (11)	0.0061 (9)	0.0179 (9)	0.0157 (9)
C34	0.0335 (10)	0.0495 (11)	0.0216 (9)	−0.0097 (9)	0.0117 (8)	0.0054 (8)
C35	0.0369 (10)	0.0474 (11)	0.0267 (9)	−0.0055 (9)	0.0195 (8)	−0.0059 (8)
C36	0.0347 (10)	0.0328 (9)	0.0258 (9)	0.0016 (8)	0.0157 (8)	−0.0005 (7)
C41	0.0298 (9)	0.0322 (9)	0.0193 (8)	−0.0040 (7)	0.0132 (7)	−0.0013 (7)
C42	0.0386 (10)	0.0320 (9)	0.0262 (9)	−0.0031 (8)	0.0157 (8)	−0.0021 (7)
C43	0.0633 (14)	0.0299 (10)	0.0306 (10)	−0.0069 (9)	0.0231 (10)	−0.0032 (8)
C44	0.0568 (14)	0.0462 (12)	0.0247 (10)	−0.0298 (11)	0.0145 (10)	−0.0085 (9)
C45	0.0367 (11)	0.0589 (14)	0.0285 (10)	−0.0172 (10)	0.0120 (9)	−0.0008 (9)
C46	0.0299 (10)	0.0447 (11)	0.0250 (9)	−0.0051 (8)	0.0123 (8)	0.0000 (8)
O11	0.0574 (9)	0.0336 (7)	0.0321 (7)	−0.0096 (7)	0.0208 (7)	0.0002 (6)
O12	0.0432 (8)	0.0277 (6)	0.0261 (7)	0.0005 (6)	0.0114 (6)	−0.0047 (5)
O13	0.1187 (18)	0.0419 (10)	0.0549 (11)	−0.0374 (10)	0.0165 (12)	−0.0063 (9)
C10	0.0319 (9)	0.0304 (9)	0.0298 (9)	−0.0035 (7)	0.0171 (8)	−0.0025 (7)
C11	0.0275 (9)	0.0357 (10)	0.0283 (9)	−0.0049 (8)	0.0134 (8)	−0.0058 (8)
C12	0.0312 (10)	0.0410 (10)	0.0318 (10)	−0.0009 (8)	0.0159 (8)	−0.0032 (8)
C13	0.0361 (11)	0.0642 (14)	0.0292 (10)	0.0020 (10)	0.0152 (9)	−0.0013 (10)
C14	0.0469 (13)	0.0823 (19)	0.0323 (12)	−0.0077 (13)	0.0097 (11)	−0.0219 (12)
C15	0.0783 (19)	0.0596 (16)	0.0462 (15)	−0.0229 (14)	0.0119 (14)	−0.0231 (13)
C16	0.0601 (15)	0.0418 (12)	0.0397 (12)	−0.0186 (11)	0.0134 (11)	−0.0099 (10)
O21	0.0328 (7)	0.0314 (7)	0.0348 (7)	0.0038 (6)	0.0102 (6)	0.0002 (6)
O22	0.0393 (7)	0.0266 (6)	0.0256 (6)	−0.0061 (5)	0.0131 (6)	−0.0041 (5)
O23	0.0414 (8)	0.0256 (7)	0.0559 (10)	0.0013 (6)	0.0139 (7)	−0.0105 (6)
C20	0.0317 (9)	0.0265 (8)	0.0225 (8)	−0.0013 (7)	0.0149 (8)	−0.0006 (7)
C21	0.0292 (9)	0.0251 (8)	0.0218 (8)	−0.0022 (7)	0.0140 (7)	−0.0011 (7)
C22	0.0341 (10)	0.0278 (9)	0.0308 (10)	−0.0012 (7)	0.0174 (8)	−0.0048 (7)
C23	0.0363 (11)	0.0398 (11)	0.0339 (10)	−0.0105 (9)	0.0173 (9)	−0.0124 (8)
C24	0.0288 (10)	0.0555 (12)	0.0269 (10)	−0.0068 (9)	0.0105 (8)	−0.0028 (9)
C25	0.0356 (11)	0.0412 (11)	0.0344 (10)	0.0079 (9)	0.0182 (9)	0.0094 (9)
C26	0.0362 (10)	0.0269 (9)	0.0282 (9)	−0.0016 (7)	0.0180 (8)	0.0005 (7)

### Geometric parameters (Å, º)

**Table:**

F1—C44	1.368 (2)	C43—H43A	0.9500
F2—C34	1.364 (2)	C44—C45	1.364 (3)
N1—C5	1.469 (2)	C45—C46	1.379 (3)
N1—C2	1.469 (2)	C45—H45A	0.9500
N1—C1	1.481 (2)	C46—H46A	0.9500
N2—C4	1.488 (2)	O11—C10	1.233 (2)
N2—C3	1.492 (2)	O12—C10	1.313 (2)
N2—H2C	0.9200	O12—H12	0.8400
N2—H2D	0.9200	O13—C16	1.349 (3)
C1—C41	1.520 (2)	O13—H13	0.8400
C1—C31	1.519 (2)	C10—C11	1.472 (3)
C1—H1A	1.0000	C11—C16	1.396 (3)
C2—C3	1.510 (2)	C11—C12	1.395 (3)
C2—H2A	0.9900	C12—C13	1.379 (3)
C2—H2B	0.9900	C12—H12A	0.9500
C3—H3A	0.9900	C13—C14	1.379 (3)
C3—H3B	0.9900	C13—H13A	0.9500
C4—C5	1.513 (2)	C14—C15	1.370 (4)
C4—H4A	0.9900	C14—H14A	0.9500
C4—H4B	0.9900	C15—C16	1.394 (3)
C5—H5A	0.9900	C15—H15A	0.9500
C5—H5B	0.9900	O21—C20	1.262 (2)
C31—C32	1.383 (2)	O22—C20	1.269 (2)
C31—C36	1.393 (2)	O23—C22	1.352 (2)
C32—C33	1.393 (3)	O23—H23	0.8400
C32—H32A	0.9500	C20—C21	1.488 (2)
C33—C34	1.363 (3)	C21—C26	1.396 (2)
C33—H33A	0.9500	C21—C22	1.404 (2)
C34—C35	1.373 (3)	C22—C23	1.393 (3)
C35—C36	1.383 (3)	C23—C24	1.373 (3)
C35—H35A	0.9500	C23—H23B	0.9500
C36—H36A	0.9500	C24—C25	1.388 (3)
C41—C42	1.386 (3)	C24—H24A	0.9500
C41—C46	1.389 (3)	C25—C26	1.373 (3)
C42—C43	1.389 (3)	C25—H25A	0.9500
C42—H42A	0.9500	C26—H26A	0.9500
C43—C44	1.376 (3)		
			
C5—N1—C2	109.10 (13)	C41—C42—C43	120.49 (19)
C5—N1—C1	111.43 (12)	C41—C42—H42A	119.8
C2—N1—C1	109.35 (13)	C43—C42—H42A	119.8
C4—N2—C3	110.84 (13)	C44—C43—C42	117.9 (2)
C4—N2—H2C	109.5	C44—C43—H43A	121.1
C3—N2—H2C	109.5	C42—C43—H43A	121.1
C4—N2—H2D	109.5	C45—C44—F1	118.4 (2)
C3—N2—H2D	109.5	C45—C44—C43	123.46 (18)
H2C—N2—H2D	108.1	F1—C44—C43	118.1 (2)
N1—C1—C41	110.80 (13)	C44—C45—C46	117.8 (2)
N1—C1—C31	111.61 (14)	C44—C45—H45A	121.1
C41—C1—C31	110.16 (13)	C46—C45—H45A	121.1
N1—C1—H1A	108.0	C45—C46—C41	121.2 (2)
C41—C1—H1A	108.0	C45—C46—H46A	119.4
C31—C1—H1A	108.0	C41—C46—H46A	119.4
N1—C2—C3	111.77 (14)	C10—O12—H12	109.5
N1—C2—H2A	109.3	C16—O13—H13	109.5
C3—C2—H2A	109.3	O11—C10—O12	121.78 (16)
N1—C2—H2B	109.3	O11—C10—C11	122.85 (17)
C3—C2—H2B	109.3	O12—C10—C11	115.37 (16)
H2A—C2—H2B	107.9	C16—C11—C12	118.86 (18)
N2—C3—C2	110.42 (13)	C16—C11—C10	119.05 (18)
N2—C3—H3A	109.6	C12—C11—C10	122.08 (17)
C2—C3—H3A	109.6	C13—C12—C11	121.03 (19)
N2—C3—H3B	109.6	C13—C12—H12A	119.5
C2—C3—H3B	109.6	C11—C12—H12A	119.5
H3A—C3—H3B	108.1	C14—C13—C12	119.3 (2)
N2—C4—C5	110.40 (14)	C14—C13—H13A	120.3
N2—C4—H4A	109.6	C12—C13—H13A	120.3
C5—C4—H4A	109.6	C13—C14—C15	121.0 (2)
N2—C4—H4B	109.6	C13—C14—H14A	119.5
C5—C4—H4B	109.6	C15—C14—H14A	119.5
H4A—C4—H4B	108.1	C14—C15—C16	120.1 (2)
N1—C5—C4	109.97 (13)	C14—C15—H15A	119.9
N1—C5—H5A	109.7	C16—C15—H15A	119.9
C4—C5—H5A	109.7	O13—C16—C15	117.8 (2)
N1—C5—H5B	109.7	O13—C16—C11	122.5 (2)
C4—C5—H5B	109.7	C15—C16—C11	119.7 (2)
H5A—C5—H5B	108.2	C22—O23—H23	109.5
C32—C31—C36	118.80 (16)	O21—C20—O22	123.02 (16)
C32—C31—C1	119.83 (16)	O21—C20—C21	118.18 (15)
C36—C31—C1	121.36 (15)	O22—C20—C21	118.80 (15)
C31—C32—C33	120.86 (18)	C26—C21—C22	118.14 (16)
C31—C32—H32A	119.6	C26—C21—C20	121.14 (15)
C33—C32—H32A	119.6	C22—C21—C20	120.72 (15)
C34—C33—C32	118.29 (18)	O23—C22—C23	117.87 (16)
C34—C33—H33A	120.9	O23—C22—C21	122.19 (16)
C32—C33—H33A	120.9	C23—C22—C21	119.94 (17)
F2—C34—C35	118.22 (19)	C24—C23—C22	120.37 (18)
F2—C34—C33	118.89 (18)	C24—C23—H23B	119.8
C35—C34—C33	122.89 (18)	C22—C23—H23B	119.8
C34—C35—C36	118.25 (18)	C23—C24—C25	120.42 (18)
C34—C35—H35A	120.9	C23—C24—H24A	119.8
C36—C35—H35A	120.9	C25—C24—H24A	119.8
C35—C36—C31	120.89 (17)	C26—C25—C24	119.40 (18)
C35—C36—H36A	119.6	C26—C25—H25A	120.3
C31—C36—H36A	119.6	C24—C25—H25A	120.3
C42—C41—C46	119.15 (17)	C25—C26—C21	121.71 (17)
C42—C41—C1	121.71 (16)	C25—C26—H26A	119.1
C46—C41—C1	119.11 (16)	C21—C26—H26A	119.1
			
C5—N1—C1—C41	172.94 (14)	F1—C44—C45—C46	178.36 (17)
C2—N1—C1—C41	−66.37 (17)	C43—C44—C45—C46	−0.8 (3)
C5—N1—C1—C31	49.78 (18)	C44—C45—C46—C41	−0.2 (3)
C2—N1—C1—C31	170.47 (13)	C42—C41—C46—C45	0.9 (3)
C5—N1—C2—C3	−59.47 (17)	C1—C41—C46—C45	179.10 (17)
C1—N1—C2—C3	178.43 (13)	O11—C10—C11—C16	3.9 (3)
C4—N2—C3—C2	−53.61 (19)	O12—C10—C11—C16	−175.59 (19)
N1—C2—C3—N2	55.98 (18)	O11—C10—C11—C12	−176.00 (18)
C3—N2—C4—C5	55.80 (18)	O12—C10—C11—C12	4.6 (3)
C2—N1—C5—C4	60.67 (18)	C16—C11—C12—C13	−0.1 (3)
C1—N1—C5—C4	−178.49 (14)	C10—C11—C12—C13	179.79 (18)
N2—C4—C5—N1	−59.57 (18)	C11—C12—C13—C14	0.0 (3)
N1—C1—C31—C32	−123.99 (17)	C12—C13—C14—C15	−0.3 (4)
C41—C1—C31—C32	112.49 (18)	C13—C14—C15—C16	0.6 (5)
N1—C1—C31—C36	57.4 (2)	C14—C15—C16—O13	179.8 (3)
C41—C1—C31—C36	−66.1 (2)	C14—C15—C16—C11	−0.6 (5)
C36—C31—C32—C33	0.9 (3)	C12—C11—C16—O13	180.0 (2)
C1—C31—C32—C33	−177.75 (17)	C10—C11—C16—O13	0.1 (4)
C31—C32—C33—C34	−0.8 (3)	C12—C11—C16—C15	0.3 (4)
C32—C33—C34—F2	−179.56 (17)	C10—C11—C16—C15	−179.5 (2)
C32—C33—C34—C35	−0.3 (3)	O21—C20—C21—C26	−176.14 (16)
F2—C34—C35—C36	−179.38 (16)	O22—C20—C21—C26	2.6 (3)
C33—C34—C35—C36	1.3 (3)	O21—C20—C21—C22	3.7 (3)
C34—C35—C36—C31	−1.3 (3)	O22—C20—C21—C22	−177.59 (16)
C32—C31—C36—C35	0.2 (3)	C26—C21—C22—O23	178.55 (17)
C1—C31—C36—C35	178.82 (16)	C20—C21—C22—O23	−1.3 (3)
N1—C1—C41—C42	−32.2 (2)	C26—C21—C22—C23	−1.0 (3)
C31—C1—C41—C42	91.76 (19)	C20—C21—C22—C23	179.13 (17)
N1—C1—C41—C46	149.63 (16)	O23—C22—C23—C24	179.95 (18)
C31—C1—C41—C46	−86.38 (19)	C21—C22—C23—C24	−0.5 (3)
C46—C41—C42—C43	−0.7 (3)	C22—C23—C24—C25	1.4 (3)
C1—C41—C42—C43	−178.80 (16)	C23—C24—C25—C26	−0.7 (3)
C41—C42—C43—C44	−0.3 (3)	C24—C25—C26—C21	−0.8 (3)
C42—C43—C44—C45	1.0 (3)	C22—C21—C26—C25	1.7 (3)
C42—C43—C44—F1	−178.13 (17)	C20—C21—C26—C25	−178.48 (17)

### Hydrogen-bond geometry (Å, º)

**Table:**

*D*—H···*A*	*D*—H	H···*A*	*D*···*A*	*D*—H···*A*
N2—H2*C*···O22^i^	0.92	2.00	2.892 (2)	162
N2—H2*D*···O21	0.92	1.87	2.742 (2)	158
O12—H12···O22	0.84	1.73	2.5654 (18)	179
O13—H13···O11	0.84	1.83	2.574 (2)	146
O23—H23···O21	0.84	1.80	2.544 (2)	146

Symmetry code: (i) −*x*+1/2, *y*+1/2, −*z*+3/2.
